# A Primer for Single-Cell Sequencing in Non-Model Organisms

**DOI:** 10.3390/genes13020380

**Published:** 2022-02-19

**Authors:** James M. Alfieri, Guosong Wang, Michelle M. Jonika, Clare A. Gill, Heath Blackmon, Giridhar N. Athrey

**Affiliations:** 1 Department of Biology, Texas A&M University, College Station, TX 77843, USA; michelle19@tamu.edu (M.M.J.); hblackmon@bio.tamu.edu (H.B.); 2 Interdisciplinary Program in Ecology and Evolutionary Biology, Texas A&M University, College Station, TX 77843, USA; giri.athrey@tamu.edu; 3 Department of Poultry Science, Texas A&M University, College Station, TX 77843, USA; 4 Department of Animal Science, Texas A&M University, College Station, TX 77843, USA; guosong.wang@tamu.edu (G.W.); clare.gill@ag.tamu.edu (C.A.G.); 5 Interdisciplinary Program in Genetics and Genomics, Texas A&M University, College Station, TX 77843, USA

**Keywords:** single-cell sequencing, non-model organism, ecology, evolution, animal science

## Abstract

Single-cell sequencing technologies have led to a revolution in our knowledge of the diversity of cell types, connections between biological levels of organization, and relationships between genotype and phenotype. These advances have mainly come from using model organisms; however, using single-cell sequencing in non-model organisms could enable investigations of questions inaccessible with typical model organisms. This primer describes a general workflow for single-cell sequencing studies and considerations for using non-model organisms (limited to multicellular animals). Importantly, single-cell sequencing, when further applied in non-model organisms, will allow for a deeper understanding of the mechanisms between genotype and phenotype and the basis for biological variation.

## 1. Introduction

Cells are the fundamental units of life, varying in form and function within and between individuals, populations, and species. The sequencing of nucleic acids within single-cells, known as single-cell sequencing (scSeq), has led to a revolution in our knowledge of the diversity of cell types at varying levels of the biological hierarchy (e.g., tissue, organ, individual), while also advancing our understanding of genotype x phenotype relationships. As in most of biology, our knowledge of humans and model organisms (e.g., Drosophila) far exceeds that of non-model or wild species, which remains a barrier to resolving how organisms function in nature. At present, this remains the case for scSeq; although scSeq is a promising and proven method, its use has been limited and its potential applications in non-model systems have yet to be realized. Nevertheless, there have been a few examples where scSeq has already been used in non-model organisms to investigate questions that are inaccessible with typical model organisms (e.g., spinal cord regeneration in some amphibians and fish [1]).

The fast pace of technological advancement in the field, requirement of organism-specific technical knowledge, and cost are all barriers to applying scSeq to non-model organisms. The first challenge for applications of scSeq in non-model organisms is its recency; since the first study published in 2009 [2], the technology has evolved rapidly, and a slower adoption in non-model organisms is to be expected. Another barrier to scSeq in non-model organisms is the need for organism-specific technical knowledge for sample collection and cell isolation protocols. For example, organism-specific protocols for mechanical and enzymatic digestion of intra- and extra-cellular structures are often required [3]. Another barrier to scSeq is cost; although scSeq is significantly cheaper since it became commercially available, it can be prohibitively expensive compared to bulk sequencing (approximately a 10-fold difference in early 2022).

This primer describes a general workflow for scSeq studies and includes considerations for using non-model organisms. Although many genomic characteristics can be investigated with scSeq (such as chromatin accessibility, methylation, and histone modifications among others), we focus on single-cell DNA sequencing (scDNA-seq) for variant calling and single-cell RNA sequencing (scRNA-seq) for expression quantification as these two approaches are foundational technologies. Importantly, researchers should also consider single-cell multiomic approaches, where multiple genomic features are investigated in the same cell [4]. Finally, we limit the scope of this review to multicellular animals. When applied in non-model organisms, scSeq will allow for a deeper understanding of the relationship between genotype and phenotype and the basis for biological variation.

## 2. Considerations When Planning a scSeq Study in Non-Model Organisms

The specific aims of a proposed study will determine the amount and type of background knowledge necessary (reference genome, cell type information, other publicly available scSeq data, etc.), the specific protocols (sample preparation, sequencing strategy, etc.), and data analysis techniques used. Indeed, scSeq projects have been performed on diverse organisms such as lizards [5], ants [6], fish [7], sponges [8], and flatworms [9,10]. Svensson et al. maintains a curated database of scSeq studies which can be filtered by organism and tissue type [11]. Broadly, the scSeq approaches we focus on are scRNA-seq and scDNA-seq. These approaches differ in that scRNA-seq approaches are concerned with quantifying and characterizing the expression of mRNA transcripts, whereas scDNA-seq approaches are concerned with quantifying and characterizing sequence variation. Protocols used in scDNA-seq and scRNA-seq approaches differ because of the molecular differences between RNA and DNA; RNA is less stable but abundant in the number of transcripts, whereas DNA is more stable but limited to the number of chromosomes present in a single cell. Researchers will need to know which broad approach will best address their aims to choose suitable methods.

### 2.1. Reference Genome or No Reference Genome?

Researchers should assess the availability and quality of the focal organism’s reference genome before embarking on a scSeq project, as many protocols rely on reference-based methods [12]. For species with a reference genome, depending on the aim and scope of the project, fragmented or poorly annotated genomes may allow for sufficient mapping to quantify the expression of major genes. However, reference genomes with limited annotation information will limit the study of gene duplicates, isoforms, novel non-coding transcripts, or genes within poorly assembled regions of the genome, as the sequencing depth of scRNA-seq usually does not allow researchers to identify novel non-coding features. For species without a reference genome, there are approaches for de novo construction of the transcriptome. For example, Sun et al. proposed a compressed k-mers group (CKG)-based approach [13]. Although this method was originally developed to address data sparsity in scRNA-seq analysis, it can be utilized for purposes such as identification of cell types as it depends on k-mers instead of gene expression profiles. Additionally, Nip et al. proposed RNA-Bloom which is a reference-free method for the construction of transcriptome to study the expression of isoforms for scRNA-seq [14]. In the foreseeable future, there will be more bioinformatics tools to analyze scSeq data that do not require reference genomes [14,15]. Despite these bioinformatic solutions, the aims and scope of a scSeq project may be constrained by the quality of the reference genome.

### 2.2. Sample Preparation for scSeq

As scSeq has become more popular, sample collection protocols and library construction have become robust and highly optimized. Today, preparing single-cell suspensions remains a key challenge faced by researchers who wish to apply this approach in non-model species [12]. With any type of scSeq, a challenge is to keep track of the thousands of cell types. Whether we are interested in DNA- or RNA-based workflows, methods to index thousands of cells are required. Currently common approaches to index are SCI-Seq [16] and SPLiT-Seq [17]. SCI-seq uses a transposase-based indexing followed by a PCR-based indexing to generate unique indexes for thousands of cells. Split-seq uses PCR-based indexing and does not require specialized equipment. Although preparing single-cell suspensions and indexing cells are challenges, strategies and protocols do exist. Below, we describe these strategies and protocols for various scSeq approaches.

#### 2.2.1. Whole-Cell Sequencing

The first strategy, whole-cell sequencing, is very similar to protocols used in human studies, where fresh samples are collected and processed to prepare single-cell suspensions [18]. To prepare single-cell suspensions, the ideal workflow is to collect and process the samples immediately to avoid unnecessary cell damage. Unlike human samples that are usually collected and processed immediately, animal specimens, particularly from field-captured individuals, may require hours of transport between the sample collection site and the lab, and in the absence of a sterile sampling environment. To overcome such challenges, several solutions have been developed to preserve tissues for up to 48 h, such as Miltenyi Biotec’s MACS Tissue Storage Solution [19]. However, it is not clear whether storing tissues in these solutions impacts features such as gene expression [20,21,22].

Once tissues arrive in the lab, generating single-cell suspensions and library construction are both time-sensitive steps. A challenge for specimens from non-model organisms is deciding the optimal tissue dissociation methods, as organism-specific protocols do not exist in most cases. Generally, all dissociation protocols consist of mechanical digestion and enzymatic digestion [23]. In the mechanical digestion stage, tissues are washed with pre-cooled PBS before they are minced into small pieces, the size of which depends on the subsequent enzymatic digestion. A cocktail of enzymes suited to the tissue types can be applied to generate the final single-cell suspensions for enzymatic digestion. Identifying the ideal combination of enzymes in the cocktail for digestion can be challenging, as cell physiology can vary across organisms. Typical enzymes used include dispase, collagenase, hyaluronidase, papain, DNase-I, accutase, and TrypLE, among others [3]. Lafzi et al. presented a list of enzymes for each tissue type in human and mouse studies, and while it is not optimal for non-model organisms, it is a starting point for similar tissues [24]. Therefore, it is recommended that preliminary data with the appropriate enzyme recipe are obtained before formal sample collection. Preliminary knowledge can be developed by collecting tissue from a similar animal and tissue in the lab and replicating the storage and time lag that target tissues will experience.

#### 2.2.2. Single-Nucleus Sequencing

Single-nucleus sequencing is an alternate strategy to achieve comparable results as whole-cell sequencing [18]. Single-nucleus sequencing is simpler and has a more flexible timeframe than whole-cell sequencing. However, one disadvantage is that genetic materials in the cytoplasm are lost. The only special handling needed for this strategy is sample collection in liquid nitrogen and storage at −80 °C.

For single-nucleus sequencing, the integrity of nuclei is critical because nucleic acids may escape disassociated nuclei, causing failure or bias in downstream sequencing. Nuclei can be extracted using either the rapid, efficient and practical [25] method or the Isolation of Pure Nuclei Using Sucrose method [26]. Neither of these methods requires harsh mechanical homogenization such as grinding with liquid nitrogen which can degrade nuclei integrity. The sucrose method is inexpensive but is time-consuming taking at least 2 h to complete. The homogenization buffer used in the sucrose method contains isosmotic sucrose which has ions such as Mg^2+^ that serve to stabilize the integrity of nuclei [27]. The viscosity of sucrose can also induce further protein degradation, again, a key factor to indicate the integrity of the nuclei membrane. On the other hand, the REAP method can extract nuclei in a relatively short amount of time (as little as two minutes) and can maintain nuclei integrity as well as protein complex composition. The REAP method has become more popular as it uses mild detergent, such as NP-40 and triton X-100, in the homogenization buffer and does not require time-consuming steps such as velocity centrifugation through a denser layer of sucrose. However, care must be taken because utilization of nonionic solutions may affect the integrity of protein complex composition. Such degradation of the protein complex may result in damage to downstream library construction and sequencing for scSeq.

### 2.3. Library Preparation and Sequencing

#### 2.3.1. scDNA-seq Library Preparation and Sequencing

DNA isolated from a single cell is the target unit for scDNA-seq [28]. The major technical challenge with scDNA-seq is that sequencing platforms require greater amounts of DNA than an individual cell contains [29]. This problem is further compounded by sequencing error rates. Confidence that a particular base being sequenced is accurate depends on its coverage, which is dependent on sequencing depth and template quantity. This is typically not a problem for bulk tissue DNA sequencing, as many copies of the genome are obtained from pooled cells. However, in a single cell, the number of available genomes is naturally constrained by the ploidy of the organism.

Whole-genome amplification attempts to overcome this limitation of insufficient genomic starting materials by amplifying the genome. However, amplification methods can be biased and mutagenic due to PCR errors, thus affecting downstream interpretation of genomic variation [30].

Single cell multiple displacement amplification (SCMDA) is a technique that amplifies the genome without using sequence-specific primers and improves the annealing of hexamer primers [31]. These considerations preserve SNP integrity by addressing cytosine-deamination artifacts and decreasing sequence-specific amplification bias. Single cell multiple displacement amplification was developed alongside a bioinformatic approach, SCcaller, which further corrects for amplification bias and can yield genome wide average coverage of ~30X, making it suitable for SNP calling. However, SCMDA may not be reliable for detecting copy number variants, because of uneven amplification across the genome, leading to variable coverage [32].

Linear Amplification via Transposon Insertion (LIANTI) is a technique that randomly fragments the genomes of single cells using transposases [33]. The DNA is transcribed, and RNA copies of the fragmented regions are made, followed by reverse transcription. This method leads to a genome-wide coverage of ~1X. Because the T7 promoter-tagged DNA fragments are amplified linearly, rather than exponentially, the read lengths are long (~10 kb). Due to the long read lengths obtained, this method is suitable for CNV identification.

Single-stranded sequencing using microfluidic reactors (SISSOR) uses microfluidics to amplify long segments of separated DNA strands, resulting in the sequencing of complementary and homologous DNA strands [34]. SNPs are identified if variant pairs are complementary within each chromosome, but different across the homologous chromosomes. This method allows for accurate SNP calling but requires separated DNA strands at the same genomic locations to be sequenced. SISSOR results in a reduced coverage of the genome, but with a low error rate.

The Linked-Reads Assay from 10X Genomics was a popular approach that used droplet microfluidics for scDNA-seq. This product provided long read sequences that could be used to phase haplotypes and identify structural variants (10X Genomics protocol CG00044). This product was replaced by a newer technology, 10X Genomics Chromium Single Cell CNV, which used a different cell capture workflow (10X Genomics protocol CG000246). 10X Genomics discontinued their genomic products due to a legal injunction, brought about by BioRad’s case of patent infringement (The University of Chicago and Bio-Rad Laboratories, INC. v. 10X Genomics, INC, 2019). However, this injunction was vacated, clearing a path for 10X Genomics to resume sale of the Chromium Single Cell CNV and Linked-Reads products (Bio-Rad Laboratories, INC. and University of Chicago v. 10X Genomics, 2020). However, at present, 10X Genomics does not have a scDNA-seq solution, so there are no high-throughput, commercially available scDNA-seq solutions.

#### 2.3.2. scRNA-seq Library Preparation and Sequencing

Like scDNA-seq, scRNA-seq is limited by the small amount of starting material. Messenger RNA isolated from a single cell is the target unit for single cell transcriptomics. Different levels of mRNA abundance are used to identify differentially expressed genes, and to assign cell types. There are many methods for scRNA-seq, and the methods vary in the type of cell isolation required, sensitivity, and amplification biases. Broadly, different high-throughput scRNA-seq workflows can be divided into how the single cells are separated, often based on the question being answered: droplet-based and microwell strategies.

Droplet-based strategies, where droplets containing single cells are produced, is a broadly available technology. Drop-seq and InDrop are two approaches that require cells to be isolated through droplet microfluidics [35,36]. These two technologies are advantageous because they are relatively inexpensive. However, Drop-seq requires custom, “lab-made” microfluidics devices which requires technical engineering expertise [37]. InDrop has a commercially available platform, but the cell and transcript capture efficiency is low [38]. These microfluidics scRNA-seq approaches are beneficial because the RNA extraction and library preparation can occur within the microfluidic chip; however, these approaches can be problematic because they require either technical expertise or they are not sensitive to the distribution of transcripts and cells in a sample.

Chromium Single Cell Gene Expression from 10X Genomics is a widely used, commercially available platform that also uses droplet-based microfluidics. Chromium uses a technology, “Gel bead in Emulsion” (GEM), which encapsulates single cells and reagents within the microfluidic chip. RNA extraction, reverse transcription, and barcoding occur in the GEMs [39]. Currently, Chromium is extensively used for scRNA-seq applications because it is sensitive to detecting both lowly expressed and highly expressed transcripts and able to capture ~65% of cells in a heterogenous sample (10X Genomics Webinar “Pushing the Boundaries of Gene Sensitivity with the Chromium Single Cell Gene Expression v3”). Chromium is widely used because it is an integrated system; different Chromium microfluidics chips can be purchased which are optimized for different cell types and sizes, or sample sizes, the chips are run through the Chromium controller with 10X reagent kits, and the resulting product is a sequencing-ready library. This straightforward workflow is advantageous because it minimizes in-house optimization and lowers the technical experience required of users. Additionally, other biological information, such as cell surface proteins and chromatin accessibility, can be obtained, making 10X Genomics Chromium compatible with integrated multi-omics. However, this integrated workflow results in high cost–single-cell suspension and library preparation typically costs $2000–$3000 per sample just for gene expression and does not include upstream (tissue collection/dissociation) and downstream (sequencing) costs. Samples can be pooled together to significantly lower the cost per cell in some cases. Because computational algorithms allow for demultiplexing samples with different genetic backgrounds, pooling samples has a great advantage in terms of cost as it uses fewer sequencing lanes and only one library construction is required. Satija et al. provide a tool to roughly calculate and compare the cost between multiplexing design and without-multiplexing design (satijalab.org/costpercell).

Microwell strategies, where microfluidics is used to partition single cells into microwells, is an alternative approach. Seq-well is one of the newer protocols using microwells, whereas this method has been employed in previous forms through Smart-seq, CEL-seq, MARS-seq, and mcSCRB-seq are four microwell-based approaches that require cells isolated onto microfluidic plates [40,41,42]. Seq-well is a microwell-based strategy that requires cells to be isolated through gravity microfluidics into sub nanoliter wells [42,43]. This technology uses few resources (PDMS array and a polycarbonate membrane in addition to basic laboratory supplies), providing a low-cost portable platform. To summarize, scSeq methods are rapidly developing and being optimized. The major limitation of scSeq is the small amount of starting material, and the resulting biases due to nucleic acid amplification. 10X Genomics Chromium is currently the most accessible solution for transcriptomics because of its high-throughput, sensitivity to the distribution of transcripts, straightforward workflow, and cell-capture efficiency.

#### 2.3.3. Spatial Transcriptomics

While scSeq enables transcriptomic profiling of thousands of cells, the position of these cells within a tissue is lost after sample preparation. Since the debut of spatial transcriptomics in 2017, the field has been pushed further by adding spatial transcriptomics to study the cellular transcriptional atlas without losing tissue context. There are several methods to perform spatial transcriptomics. Popular methods can be grouped into two approaches: (1) using in situ capture, which relies on barcoded bead arrays to capture mRNA molecules with oligo tails (e.g., 10X spatial transcriptomics visium); (2) using fluorescence in situ hybridization such as Slide-seq. Each method has their own advantage such as the 10X visium can provide faster and more robust results while Slide-seq can provide a higher resolution. Because spatial transcriptomics cannot provide transcriptome profiling at single-cell resolution as each capture spot represents an area with ~10 cells depending on the tissue type, computational methods have been developed to combine scRNA-seq and spatial transcriptomics to compensate for the loss of resolution in spatial transcriptomics [44,45,46]. Additionally, there are software packages such as Spotlight [47], which can combine unpaired spatial transcriptomics data with scRNA-seq (i.e., data from the same tissue type but not necessarily from the same tissue block).

### 2.4. Data Analysis

Unique considerations are required when analyzing scSeq data due to the nature of sparse data dropout events and the potential for stochastic gene expression. Genetically identical cells may show different levels of gene expression caused by the nature of gene expression being determined through a Poisson process. These challenges are exacerbated for non-model organisms without high-quality reference genomes. These unique challenges add to variation among both technical and biological replicates. In the case of scRNA-seq, it is particularly challenging to distinguish technical variation (transcripts are not detected in some samples) and biological variation (differences in transcript abundances among samples). For single-cell DNA sequencing, the major limitation is data dropout events due to low-coverage sequencing and amplification biases. For example, parsing technical variation due to amplification bias apart from low-expressed transcripts or rare alleles may result in inflation of cell-to-cell variability.

Depending on the data analysis method, preprocessing of data may be required before further computational analysis [48]. Two important considerations for preprocessing are normalization and imputation to limit inherent technical bias. Normalization of data attempts to limit the effects of technical variation while preserving actual biological heterogeneity present in the scSeq data (reviewed in [49]). Additionally, imputation methods can help reduce bias from technical variation and dropout. Machine learning and deep learning imputation methods show promise in their power to distinguish biological effects from the many sources of noise and bias inherent in scSeq data [50]. However, different imputation methods usually rely on different assumptions (e.g., Unique Molecular Identifier (UMI)-based or full-length transcript) and may not be appropriate for the specific studies [50]. A thorough consideration for the choice of imputation method should be carried out prior to downstream analysis. Once the necessary preprocessing is complete, data analysis can begin.

Depending on the question being answered, after preprocessing scRNA-seq data, several analytical and statistical methods can be applied. Two common goals with single-cell RNA data are cell-level analysis and gene-level analysis [51]. With cell-level analysis, many researchers want to identify or hierarchically cluster cell subpopulations within a given biological condition. Many clustering methods are available to researchers that are developed specifically for scRNA-seq data and more broadly applicable algorithms (reviewed in [52]). For gene-level analyses, researchers may want to identify differentially expressed genes on a single-cell scale. Similar to methods for cell-level analysis, there are many tools to help identify differential gene expression in scRNA-seq data that were developed both specifically for single-cell analysis or bulk RNA-seq data (reviewed in [53]). Altogether, these considerations and methods allow for the appropriate handling of scRNA-seq and data analysis in scRNA-seq research.

A typical scSeq analysis includes the following steps: (1) raw data pre-processing, where raw sequencing reads are demultiplexed and then filtered based on unique feature counts, total unique molecules detected and percentage of reads mapped to mitochondria; (2) data normalization, such as global log transformation of the gene expression; (3) linear dimensional reduction based on highly variable features from cell-to-cell, where this step determines the dimensionality of the dataset; (4) non-linear dimensional reduction that uses previously determined clusters to visualize and explore the dataset; and (5) identification of differentially expressed features and cell type assignment which is based on the expression of biomarkers of each cell type. Additional analyses including trajectory prediction and integration of different omics also can be applied.

Well-developed software suites for analyzing scSeq data such as Seurat [45,46] and Scanpy (Wolf et al., 2018) were developed. These packages have functions such as read mapping, QC, and downstream analyses that allow for the exploration of scSeq data in one place. Seurat was originally developed to build the spatial map of gene expression in R, with the most recent version adding support for analyzing multi-omics data obtained from most scSeq approaches. Scanpy was developed as a python package, and provides numerous functions for data visualization, as well as analyses such as the trajectory inference [44]. There are built-in functions in Scanpy to convert Seurat datasets into the format Scanpy can read, adding convenience and flexibility to the scSeq analysis.

For cross-species single-cell studies, the workflow can be extended based on the identification of cells of interest using cell-type-specific biomarkers from different species [54,55]. By defining a set of homologous genes across species, cross-species comparison of gene expression can be achieved to study problems such as the expression pattern of core genes of the cells. For closely related species, commonly used methods such as STAR can be applied to identify a set of homologous genes across species; there are also programs such as SAMap [56] to build a homologous gene set between distant species that is usually a challenge.

## 3. Examples of Research Questions Suited to scSeq Approaches in Non-Model Organisms

### 3.1. Dosage Compensation

Dosage compensation describes the biological phenomenon that produces equivalent expression between a gene located on the X or Z sex chromosome and its ancestral autosomal gene [57]. Equal gene expression is often obtained through inactivation of one copy of the X or Z chromosome in the homogametic sex, followed by upregulation of the X or Z chromosome in both sexes. However, many species exhibit some intermediate degree of dosage compensation with some genes being compensated and others escaping compensation [58]. Variation in gene expression among cells and tissues may suggest variation in the necessity of dosage compensation; if a gene is more broadly expressed, then dosage compensation will be more likely to be necessary [59]. Because scSeq provides the platform necessary to determine differential expression within and across tissues, this platform is uniquely suited to determine if intra-organism dosage requirements can explain the evolution of dosage compensation.

### 3.2. Meiotic Sex Chromosome Inactivation

Meiotic sex chromosome inactivation (MSCI) refers to the transcriptional silencing of genes located on sex chromosomes within germ cells [60]. MSCI appears to be necessary for spermatogenesis and is conserved across distant taxa [61]. However, the reason for its ubiquity remains unclear. One hypothesis is that MSCI protects against segregation distortion caused by meiotic drive. An alternative hypothesis is that MSCI is a result of sexual antagonism. scSeq will be necessary to distinguish between these hypotheses because MSCI occurs on a single-cell level and can distinguish if MSCI is occurring or not [62]. This can be achieved by evaluating the expression of certain pathways including the DNA damage-recognition and repair pathway, as well as components of the synaptonemal complex called SCP3 using scRNA-seq, or by measuring histone modifications like the phosphorylation of histone H2AFX of the sex chromosome [60].

### 3.3. Applications in Livestock Research

Livestock research can benefit greatly from scSeq, largely through improving our understanding of reproductive traits. A unique characteristic of reproductive traits is that the performance is fundamentally reflected by only a few cells, such as different stages of oocytes, and supporting cells such as granulosa cells. As there are many factors to determine the performance of reproduction at different developmental stages, traditional research methodology such as bulk RNA-sequencing may introduce noise from supporting cells. Normal procedures to quantify gene expression usually require steps to remove or isolate target cells such as oocytes. For example, the primary oocyte is more difficult to isolate from the secondary oocyte [63]. Besides, cells like granulosa cells (GCs) are usually impossible to completely isolate from the rest of the tissues and the role is crucial in oocyte maturation [64]. Investigation of these cells at different developmental stages is important to evaluate or predict reproductive success.

An important feature of scSeq is to identify cell types without isolating and purifying target cells. scSeq also provides an opportunity to study interactions between cell types. For example, it can be used to study the oocytes and supporting cells that play a crucial role shaping reproduction. scSeq, especially scRNA-seq, has been widely applied to human reproduction research. It has been extensively used to study the complex gene expression network in multiple cell types such as primordial germ cells [65,66]. For example, one study applied scRNA-seq to construct the cell atlas of testis in humans, enabling the identification of the role of testosterone in reversing two transcriptional states of pre-pubertal Sertoli cells [66]. In another study, scRNA-seq was used to perform a transcriptome analysis on placenta cells during early pregnancy, which enabled identification of regulatory responses that may minimize the immune responses to mothers [67]. Similar research methodologies can be used in livestock research. Based on this, scSeq has great potential to improve reproductive traits in livestock.

Overall, these examples illustrate how scSeq can be applied in non-model organisms help us expand our understanding of the genetic or functional basis of traits, which have relevance for foundational knowledge as well as for practical applications that benefit humanity.

## 4. Perspective

Although there are difficulties associated with scSeq for non-model organisms, we argue that this is the direction the field must go in to accurately infer biological processes from genomic variation and to build a more mechanistic link between genotype and phenotype. Importantly, work with non-model organisms requires substantial evaluation of pre-existing resources, such as the availability and quality of a reference genome, protocols to isolate and digest cell types of interest, and compatibility with bioinformatic workflows. Non-model organisms may be the most suitable for answering specific basic and applied questions. Overall, scSeq technologies, when applied to non-model organisms, may reveal unique biological processes and further our understanding of the link between genotype and phenotype.

Looking into the future we can think about the eventual destination for scSeq technologies for non-model organisms. For example, in model organisms including humans, major initiatives are underway to develop cell atlases for tissues, organs, and the whole body through developmental stages, for example the Tabula Sapiens [68]. Similar Tabula projects for other species are also emerging [69]. The overarching goals for these atlas projects are to develop comprehensive, multiomics-based, developmental trajectories for cell lineages in complex organisms. For non-model organisms, this would be an obvious future objective, but before we can plan for these, multiple technological and technical hurdles need to be overcome. However, we believe that this will be the ultimate frontier for non-model organism research.

## Data Availability

Not applicable.
